# Supplementary material for: An intrinsically interpretable neural network architecture for sequence-to-function learning
Source: Bioinformatics. 2023 Jun 30;39(Suppl 1):i413–22. doi: 10.1093/bioinformatics/btad271 (PMC10311317; doi:10.1093/bioinformatics/btad271)
Supplement: btad271_Supplementary_Data [file btad271_supplementary_data.zip › supplementary/Chikina.288.sup.pdf]

model names	abT	B	gdT	innate.lym	myeloid	stem	stroma	T.act
base model(frozen kernels)	0.196 ± 0.003	0.149 ± 0.005	0.145 ± 0.004	0.163 ± 0.006	0.273 ± 0.004	0.108 ± 0.005	0.235 ± 0.004	0.142 ± 0.005
base model(unfrozen kernels)	0.240 ± 0.003	0.210 ± 0.005	0.180 ± 0.004	0.195 ± 0.006	0.326 ± 0.004	0.139 ± 0.005	0.275 ± 0.004	0.174 ± 0.005
base+attention(frozen kernels)	0.202 ± 0.003	0.154 ± 0.005	0.151 ± 0.004	0.167 ± 0.006	0.279 ± 0.004	0.113 ± 0.006	0.239 ± 0.004	0.146 ± 0.005
base+attention(unfrozen kernels)	0.237 ± 0.003	0.206 ± 0.004	0.178 ± 0.004	0.193 ± 0.006	0.322 ± 0.004	0.139 ± 0.004	0.272 ± 0.003	0.171 ± 0.006
base+attention+interaction(frozen kernels)	0.236 ± 0.004	0.190 ± 0.005	0.180 ± 0.005	0.201 ± 0.007	0.313 ± 0.005	0.142 ± 0.006	0.270 ± 0.005	0.171 ± 0.006
base+attention+interaction(unfrozen kernels)	0.269 ± 0.004	0.242 ± 0.006	0.206 ± 0.007	0.227 ± 0.007	0.351 ± 0.007	0.168 ± 0.006	0.297 ± 0.005	0.194 ± 0.008
base+interaction(frozen kernels)	0.229 ± 0.018	0.177 ± 0.024	0.175 ± 0.018	0.196 ± 0.018	0.307 ± 0.021	0.138 ± 0.013	0.263 ± 0.023	0.168 ± 0.014
base+interaction(unfrozen kernels)	$\mathbf{0.274 \pm 0.015}$	$\mathbf{0.246 \pm 0.024}$	$\mathbf{0.211 \pm 0.015}$	$\mathbf{0.232 \pm 0.016}$	$\mathbf{0.358 \pm 0.015}$	$\mathbf{0.173 \pm 0.012}$	$\mathbf{0.303 \pm 0.013}$	$\mathbf{0.201 \pm 0.013}$
AI-TAC	0.208 ± 0.006	0.135 ± 0.008	0.165 ± 0.009	0.168 ± 0.008	0.313 ± 0.006	0.103 ± 0.006	0.259 ± 0.006	0.160 ± 0.009
Lasso Regression	0.120 ± 0.008	0.091 ± 0.007	0.087 ± 0.008	0.102 ± 0.006	0.175 ± 0.005	0.061 ± 0.006	0.162 ± 0.008	0.087 ± 0.006

Table S1: We computed the R^2 values for each model, and all models underwent training and testing using the identical 10 data folds.

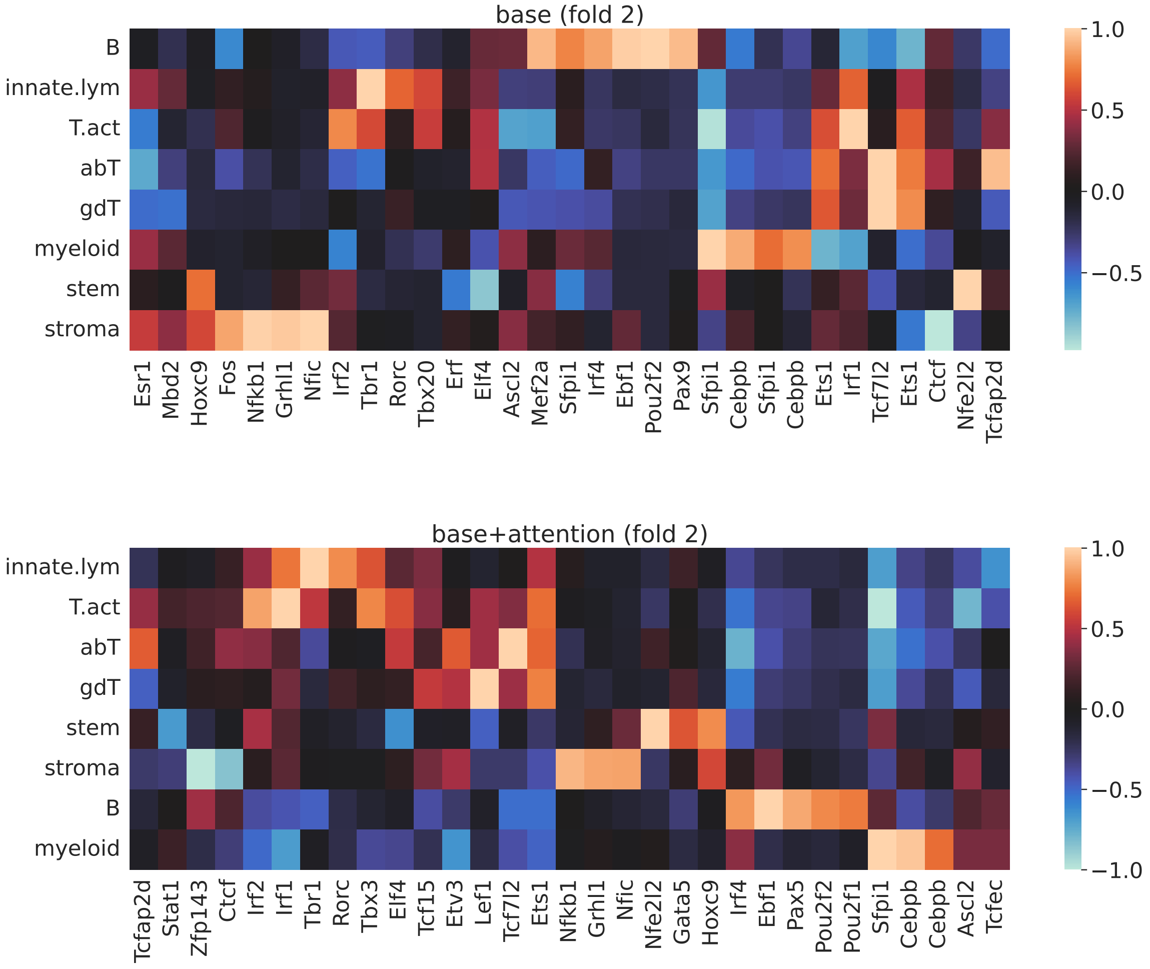

Figure S1: The final-layer coefficients captured by different versions of the tiSFM model.

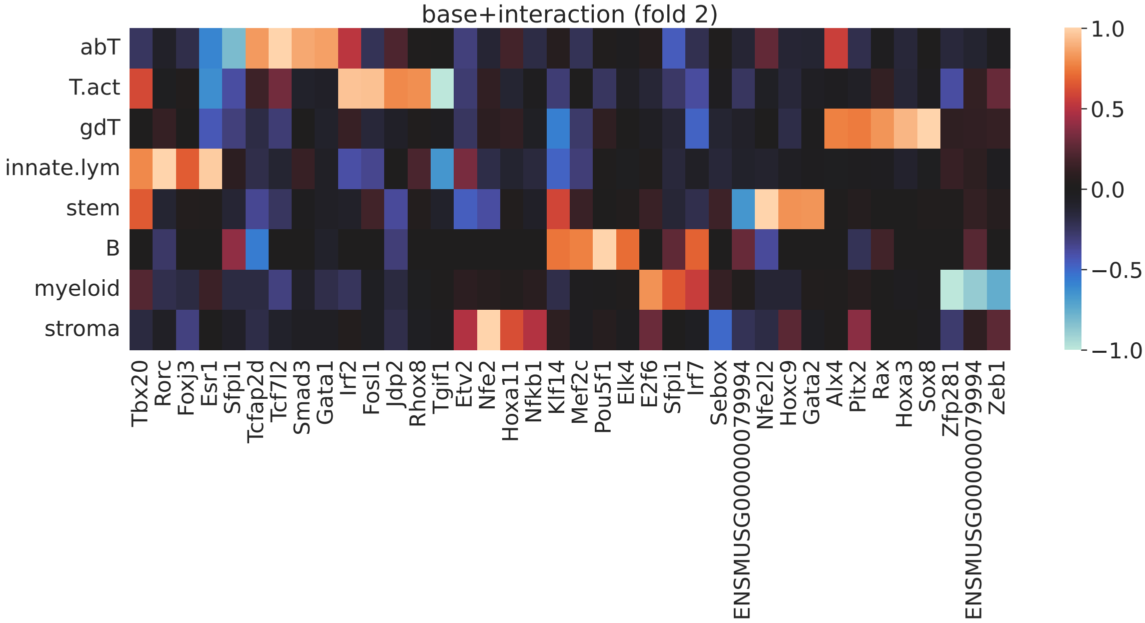

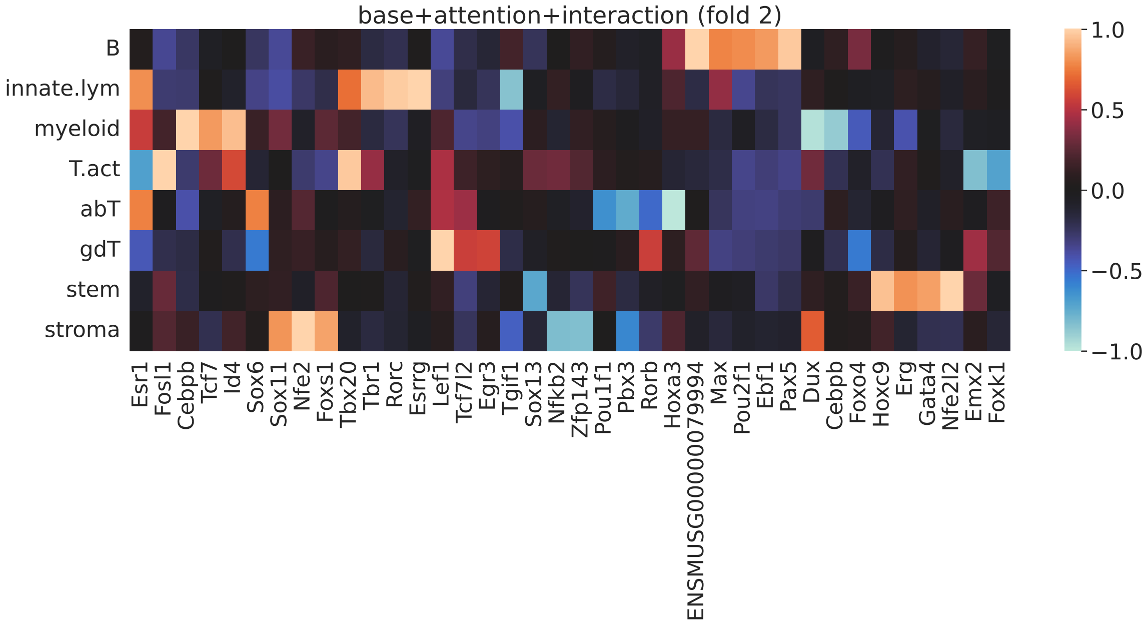

Figure S1: The final-layer coefficients captured by different versions of the tiSFM model (cont.).

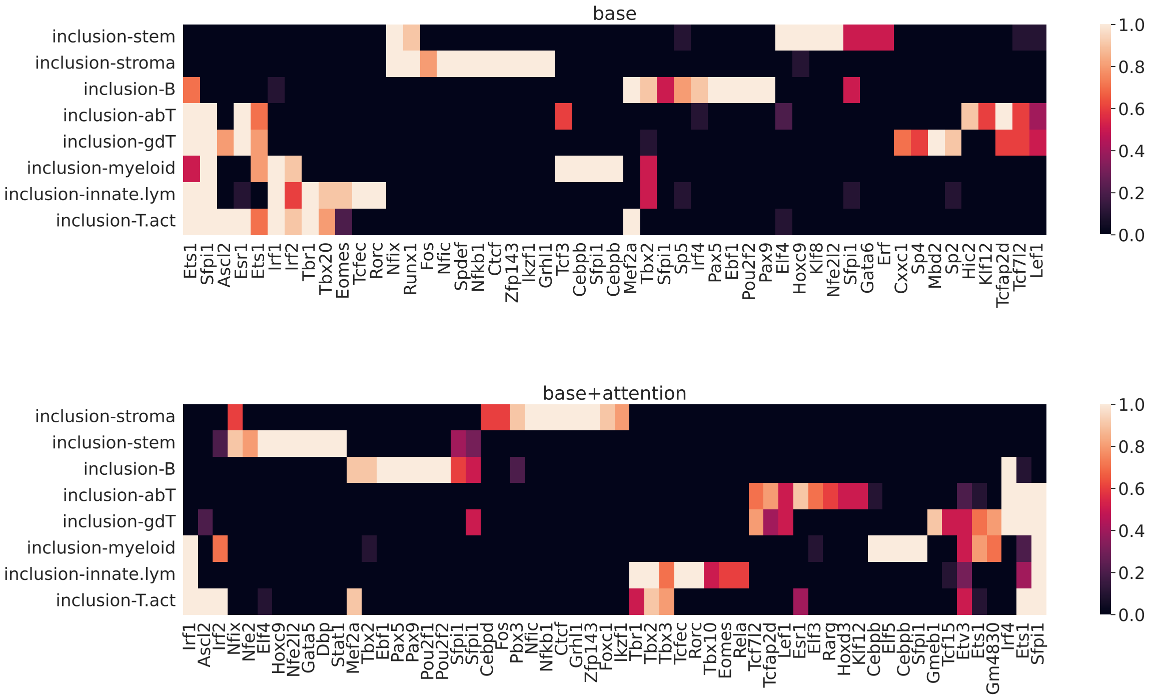

Figure S2: Heatmaps dictating inclusion across the total amount of folds tested.

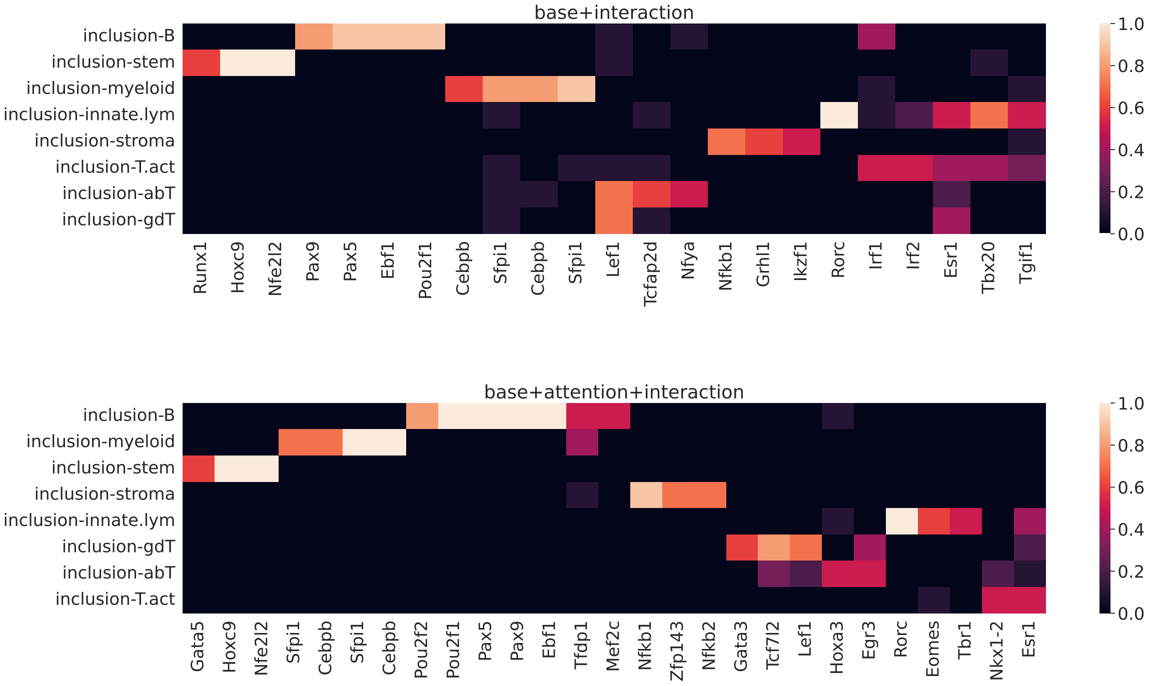

Figure S2: Heatmaps dictating inclusion across the total amount of folds tested (cont.).

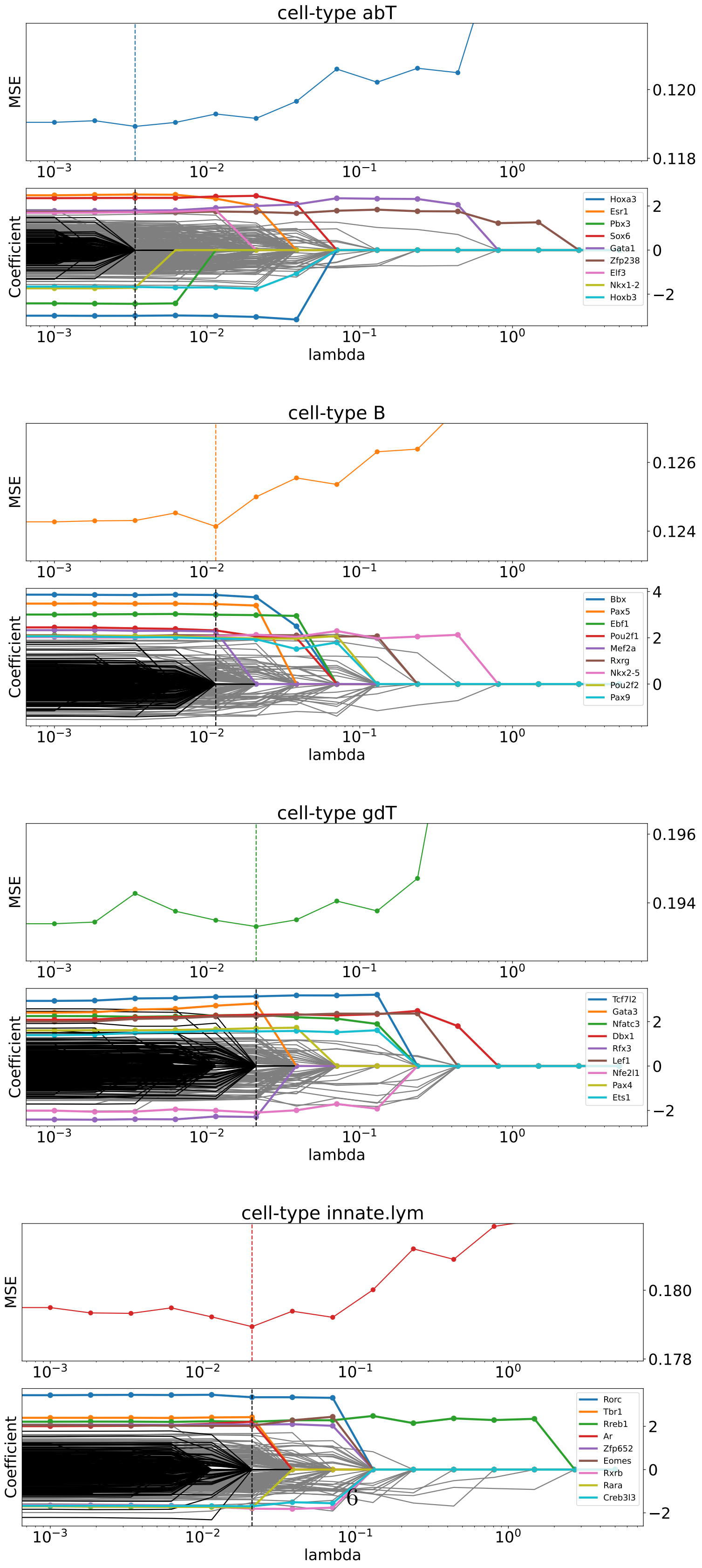

Figure S3: All cell-types and their corresponding PATH plots.

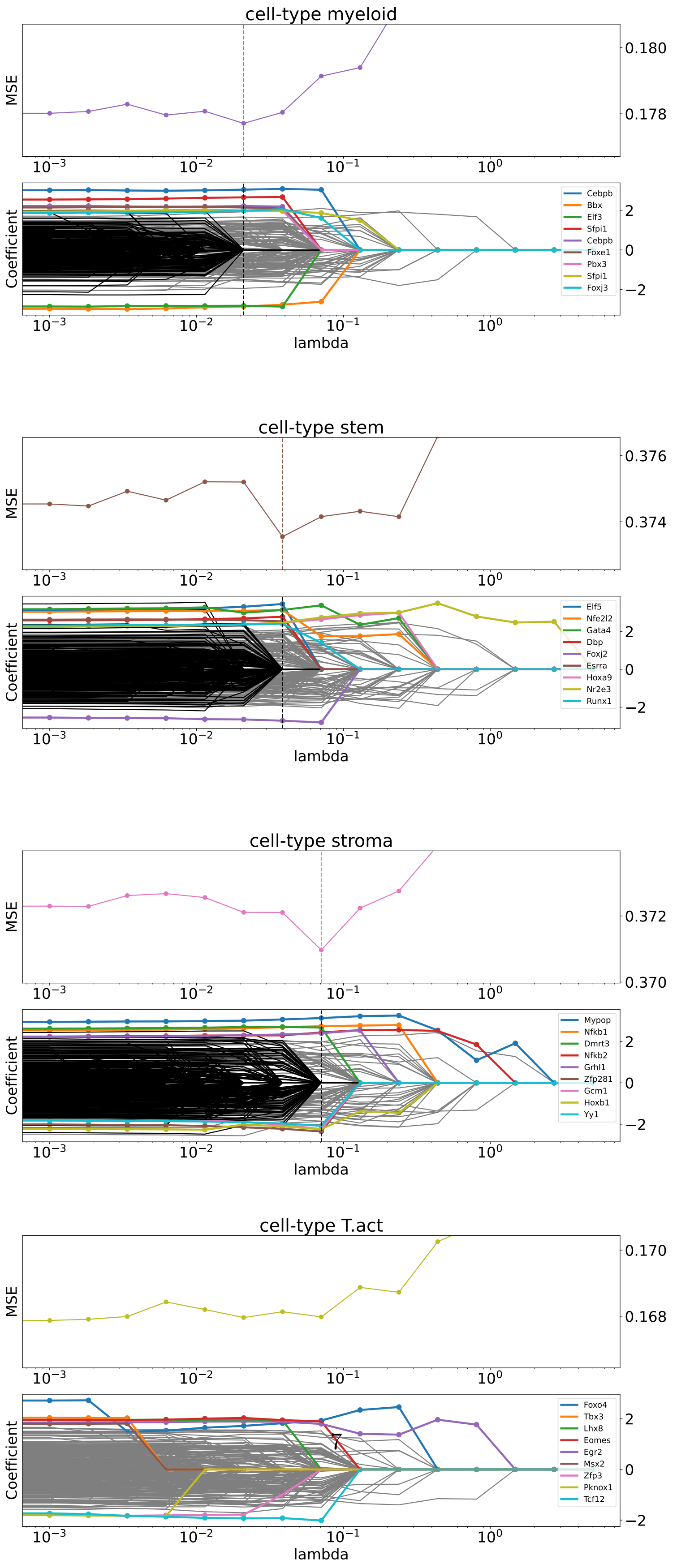

Figure S3: All cell-types and their corresponding PATH plots (cont.).

transition	variance	MSE	R2
LTHSC.34+.BM-LTHSC.34-.BM	2.07	2.02	0.02
STHSC.150-.BM-LTHSC.34+.BM	1.95	1.91	0.02
MMP3.48+.BM-STHSC.150-.BM	1.82	1.81	0.01
MMP4.135+.BM-STHSC.150-.BM	1.47	1.43	0.02
proB.CLP.BM-MMP4.135+.BM	1.40	1.34	0.05
proB.FrA.BM-proB.CLP.BM	1.16	1.15	0.01
proB.FrBC.BM-proB.FrA.BM	2.10	1.86	0.11
preB.FrD.BM-proB.FrBC.BM	1.93	1.89	0.02
B.FrE.BM-preB.FrD.BM	1.97	1.94	0.02
B1b.PC-B.FrE.BM	2.43	2.37	0.02
B.Sp-B.FrE.BM	2.20	2.17	0.02
B.Fem.Sp-B.FrE.BM	2.43	2.34	0.04
B.T1.Sp-B.FrE.BM	1.86	1.84	0.01
B.T2.Sp-B.T1.Sp	2.02	1.98	0.02
B.T3.Sp-B.T2.Sp	2.05	2.04	0.00
B.Fo.Sp-B.T3.Sp	1.93	1.93	-0.00
B.MZ.Sp-B.FrE.BM	2.32	2.27	0.02
B.mem.Sp-B.Fo.Sp	2.08	2.06	0.01
B.GC.CC.Sp-B.Fo.Sp	2.24	2.18	0.03
B.GC.CB.Sp-B.GC.CC.Sp	1.63	1.60	0.02
B.PB.Sp-B.Fo.Sp	2.73	2.62	0.04
B.PC.Sp-B.PB.Sp	2.33	2.27	0.03
B.PC.BM-B.PC.Sp	2.42	2.35	0.03
preT.DN1.Th-MMP4.135+.BM	1.34	1.29	0.03
preT.DN2a.Th-preT.DN1.Th	1.20	1.17	0.02
preT.DN2b.Th-preT.DN2a.Th	1.51	1.40	0.07
preT.DN3.Th-preT.DN2b.Th	1.56	1.54	0.01
T.DN4.Th-preT.DN3.Th	1.66	1.63	0.02
T.ISP.Th-T.DN4.Th	1.71	1.71	0.00
T.DP.Th-T.ISP.Th	2.01	1.97	0.02
T.4.Th-T.DP.Th	2.19	2.03	0.07
T.8.Th-T.DP.Th	2.26	2.06	0.09
T.4.Nve.Sp-T.4.Th	2.10	2.08	0.01
Treg.4.25hi.Sp-T.4.Th	2.35	2.28	0.03
NKT.Sp-T.4.Th	2.53	2.43	0.04
Treg.4.FP3+.Nrplo.Co-T.4.Nve.Sp	2.67	2.54	0.05
T.4.Sp.aCD3+CD40.18hr-T.4.Nve.Sp	2.18	2.11	0.03
T.8.Nve.Sp-T.8.Th	1.80	1.77	0.02
T8.TN.P14.Sp-T.8.Nve.Sp	1.70	1.68	0.01
T8.TE.LCMV.d7.Sp-T.8.Nve.Sp	2.35	2.26	0.04
T8.MP.LCMV.d7.Sp-T.8.Nve.Sp	2.21	2.13	0.03
T8.IEL.LCMV.d7.Gut-T.8.Nve.Sp	2.40	2.32	0.03
T8.Tcm.LCMV.d180.Sp-T8.MP.LCMV.d7.Sp	2.09	2.06	0.01
T8.Tem.LCMV.d180.Sp-T8.MP.LCMV.d7.Sp	2.20	2.19	0.00
Tgd.g2+d17.24a+.Th-T.DN4.Th	1.90	1.85	0.02
Tgd.g2+d1.24a+.Th-T.DN4.Th	2.01	1.96	0.02
Tgd.g1.1+d1.24a+.Th-T.DN4.Th	1.93	1.78	0.08
Tgd.Sp-T.DN4.Th	2.22	2.09	0.06
Tgd.g2+d17.LN-Tgd.g2+d17.24a+.Th	3.00	2.84	0.05
Tgd.g2+d1.LN-Tgd.g2+d1.24a+.Th	2.46	2.40	0.02
Tgd.g1.1+d1.LN-Tgd.g1.1+d1.24a+.Th	2.24	2.12	0.05
NK.27+11b-.Sp-MMP4.135+.BM	2.97	2.50	0.16
NK.27+11b+.Sp-NK.27+11b-.Sp	1.56	1.54	0.01
NK.27-11b+.Sp-NK.27+11b+.Sp	1.82	1.82	-0.00
NK.27+11b-.BM-MMP4.135+.BM	2.85	2.38	0.16
NK.27+11b+.BM-NK.27+11b-.BM	1.47	1.47	-0.00
NK.27-11b+.BM-NK.27+11b+.BM	1.83	1.79	0.02
ILC2.SI-MMP4.135+.BM	3.16	2.67	0.15
ILC3.NKp46-CCR6-.SI-MMP4.135+.BM	3.22	2.67	0.17
ILC3.CCR6+.SI-MMP4.135+.BM	3.14	2.60	0.17
ILC3.NKp46+.SI-MMP4.135+.BM	3.23	2.71	0.16
GN.BM-MMP3.48+.BM	3.52	3.22	0.09
GN.Sp-GN.BM	2.26	2.23	0.01
GN.Thio.PC-GN.Sp	2.06	2.04	0.01
DC.103+11b+.SI-MMP3.48+.BM	3.15	2.77	0.12
DC.103+11b-.SI-MMP3.48+.BM	3.15	2.70	0.14
DC.8+.Sp-MMP3.48+.BM	3.28	2.81	0.14
DC.4+.Sp-MMP3.48+.BM	2.99	2.68	0.10
DC.pDC.Sp-MMP3.48+.BM	2.98	2.68	0.10
Mo.6C+II-.Bl-MMP3.48+.BM	3.09	2.72	0.12
Mo.6C-II-.Bl-MMP3.48+.BM	3.26	2.91	0.11
MF.226+II+480lo.PC-MMP3.48+.BM	3.25	2.73	0.16
MF.RP.Sp-MMP3.48+.BM	3.35	2.97	0.12
MF.PC-MMP3.48+.BM	3.25	2.81	0.14
MF.Fem.PC-MMP3.48+.BM	3.41	2.99	0.12
MF.SI-Mo.6C+II-.Bl	2.12	2.03	0.04
MF.microglia.CNS-Mo.6C+II-.Bl	2.90	2.72	0.06
MF.Alv.Lu-Mo.6C-II-.Bl	2.35	2.27	0.03
MF.pIC.Alv.Lu-MF.Alv.Lu	1.54	1.53	0.01
NKT.Sp.LPS.3hr-NKT.Sp	2.12	2.11	0.00
NKT.Sp.LPS.18hr-NKT.Sp.LPS.3hr	1.99	1.97	0.01
NKT.Sp.LPS.3d-NKT.Sp.LPS.18hr	1.95	1.94	0.01

Table S2: The complete results for tree-diff dataset. The variance corresponds to the variance in the output while the R2 and MSE are a measure of prediction accuracy

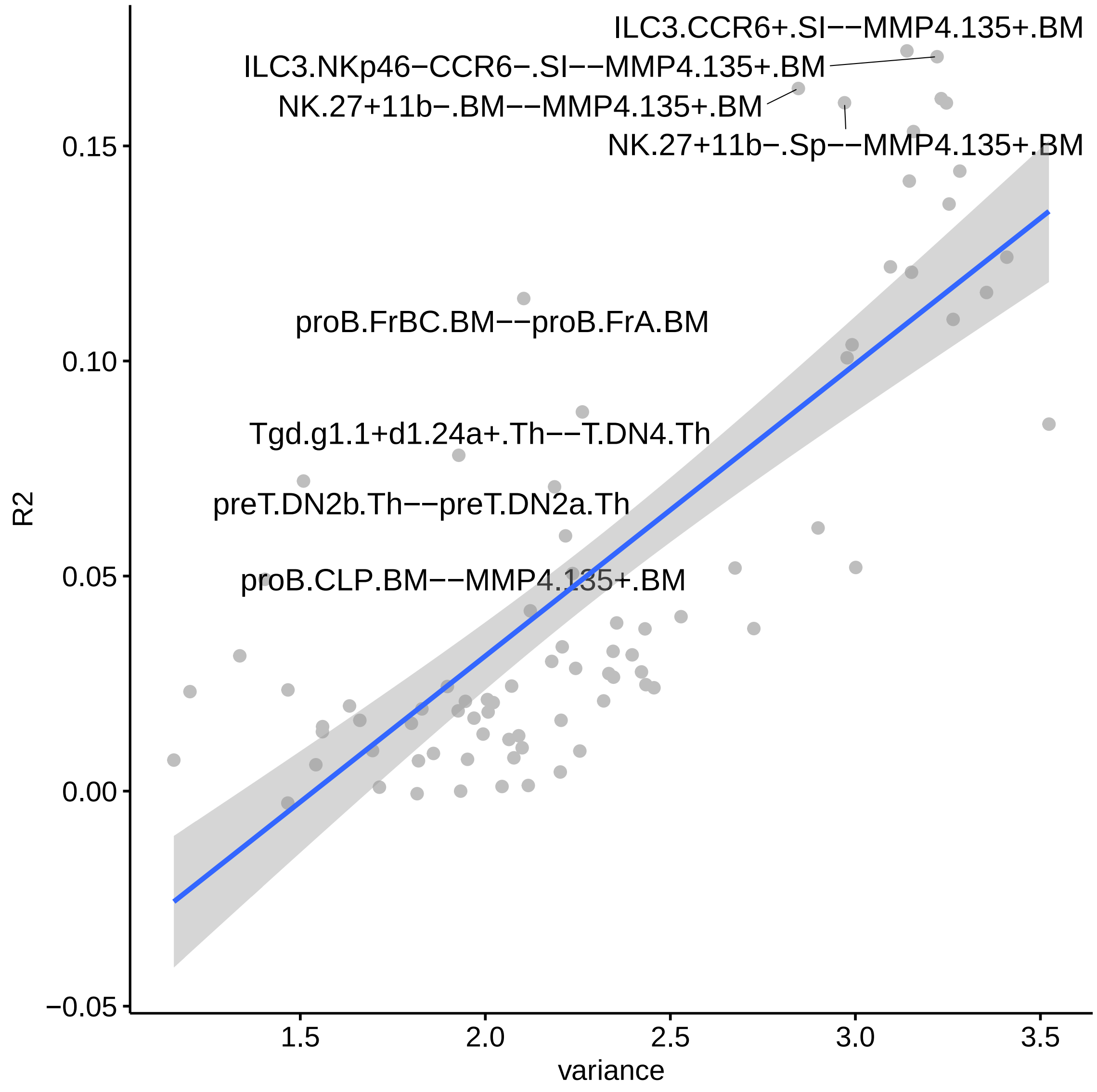

Figure S4: Relationship between the variance in OCR activity difference and its predictability as measured by R^2. Generally the two are highly correlated though some transitions are clear outliers.
