# Supplementary figures and images for: An intrinsically interpretable neural network architecture for sequence-to-function learning
Source: Bioinformatics. 2023 Jun 30;39(Suppl 1):i413–22. doi: 10.1093/bioinformatics/btad271 (PMC10311317; doi:10.1093/bioinformatics/btad271)

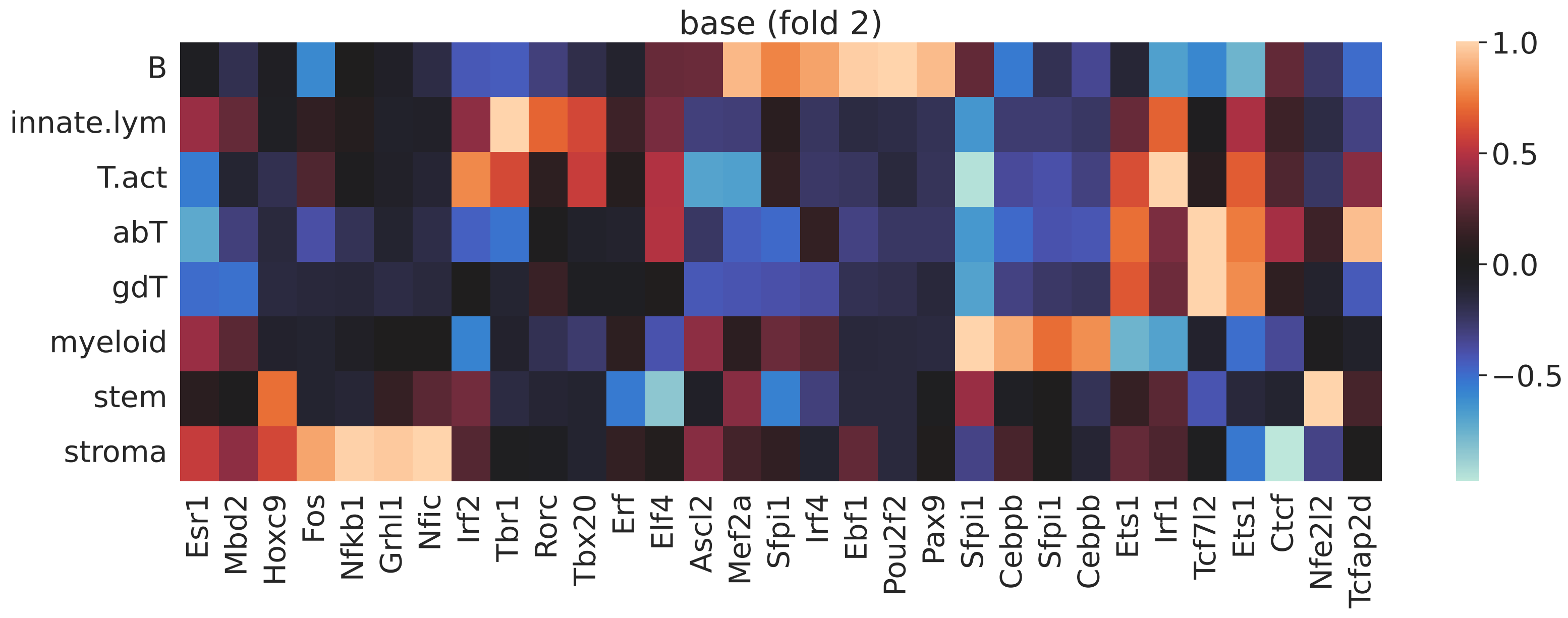

Supplement: btad271_Supplementary_Data [file btad271_supplementary_data.zip › supplementary/figures/Chikina.288.sup.1.pdf]

base+attention (fold 2)

innate.lym  
T.act  
abT  
gdT  
stem  
stroma  
B  
myeloid

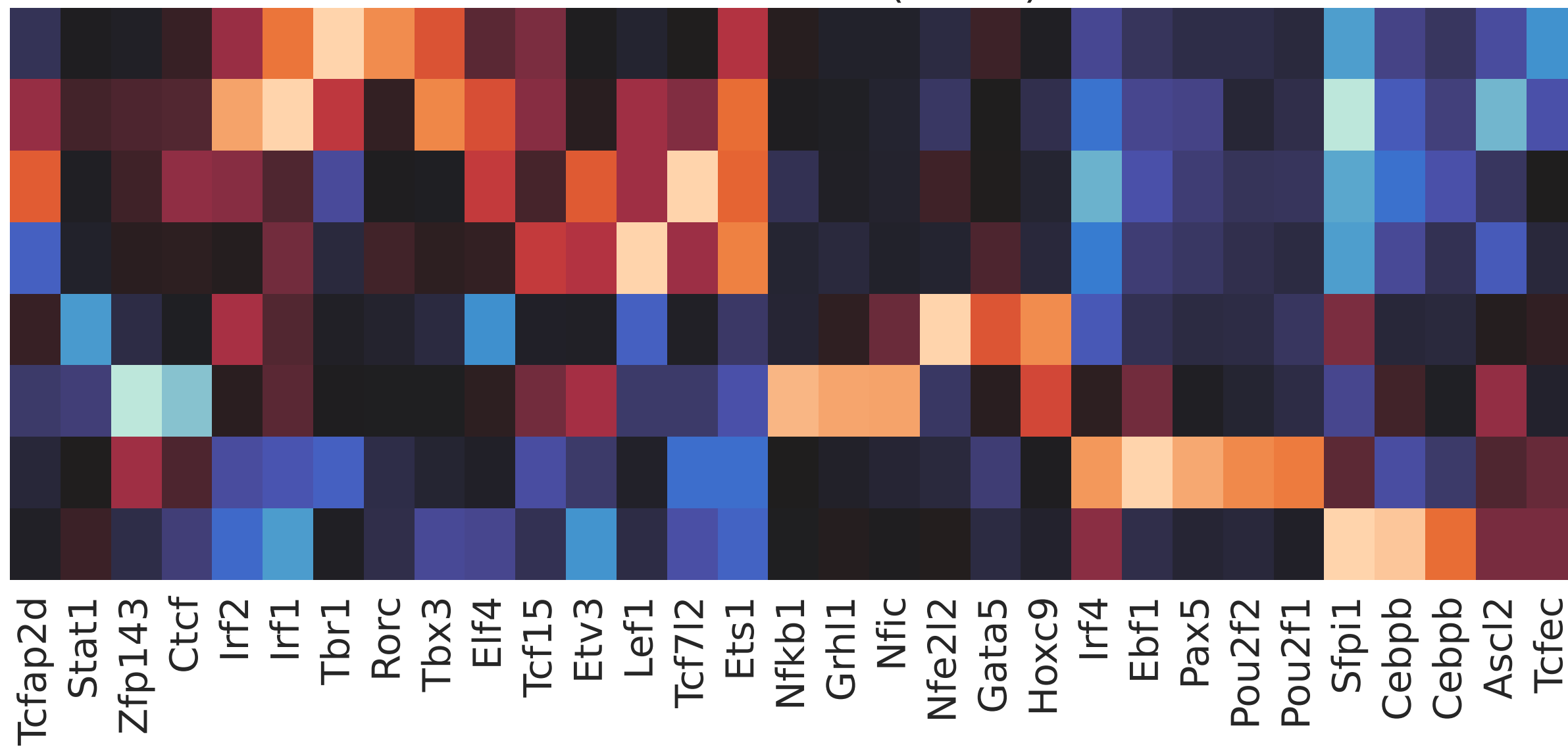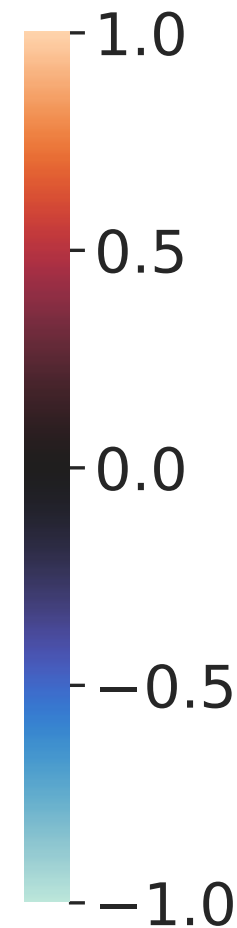

Supplement: btad271_Supplementary_Data [file btad271_supplementary_data.zip › supplementary/figures/Chikina.288.sup.2.pdf]

base+interaction (fold 2)

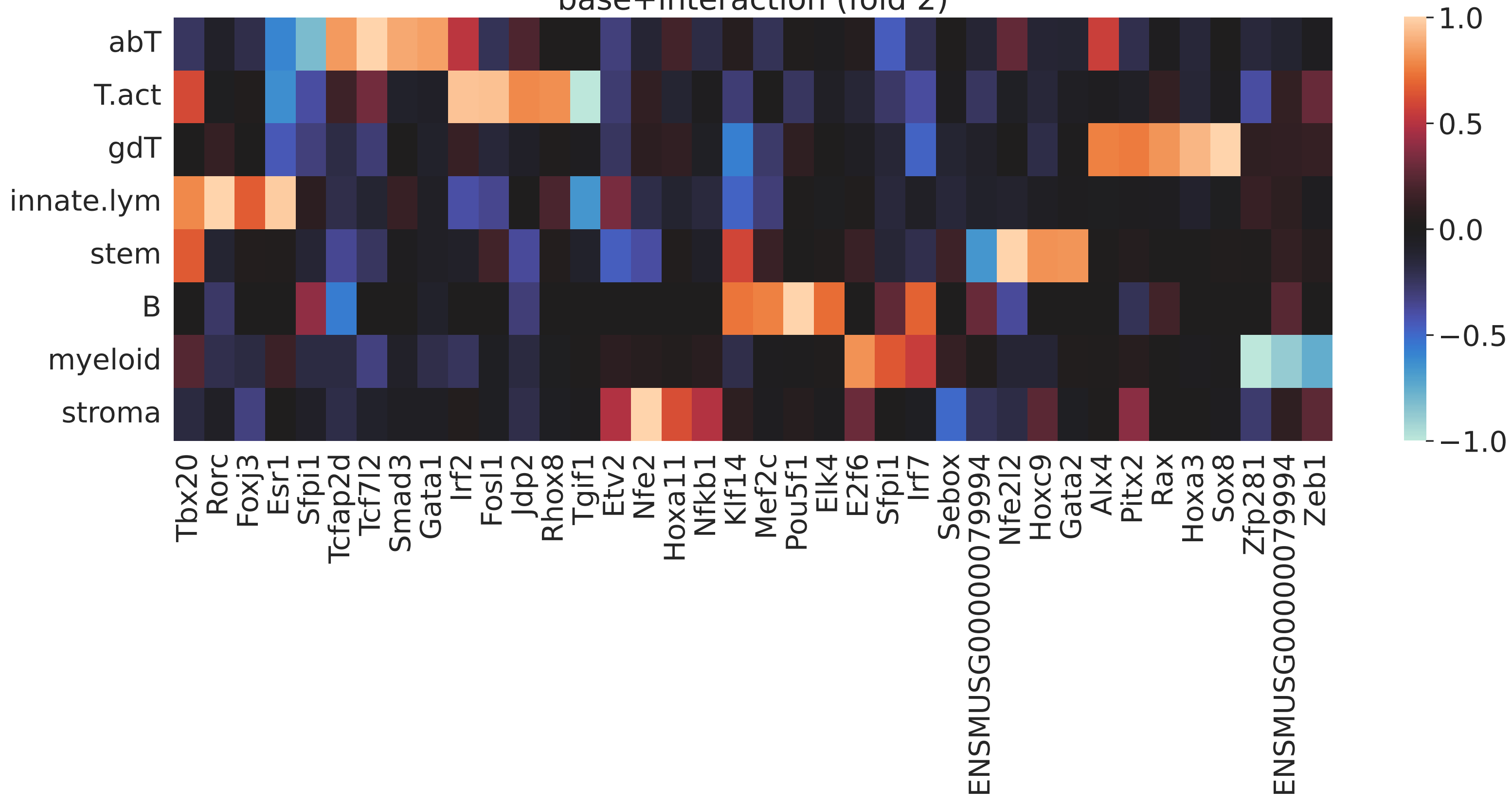

Supplement: btad271_Supplementary_Data [file btad271_supplementary_data.zip › supplementary/figures/Chikina.288.sup.3.pdf]

base+attention+interaction (fold 2)

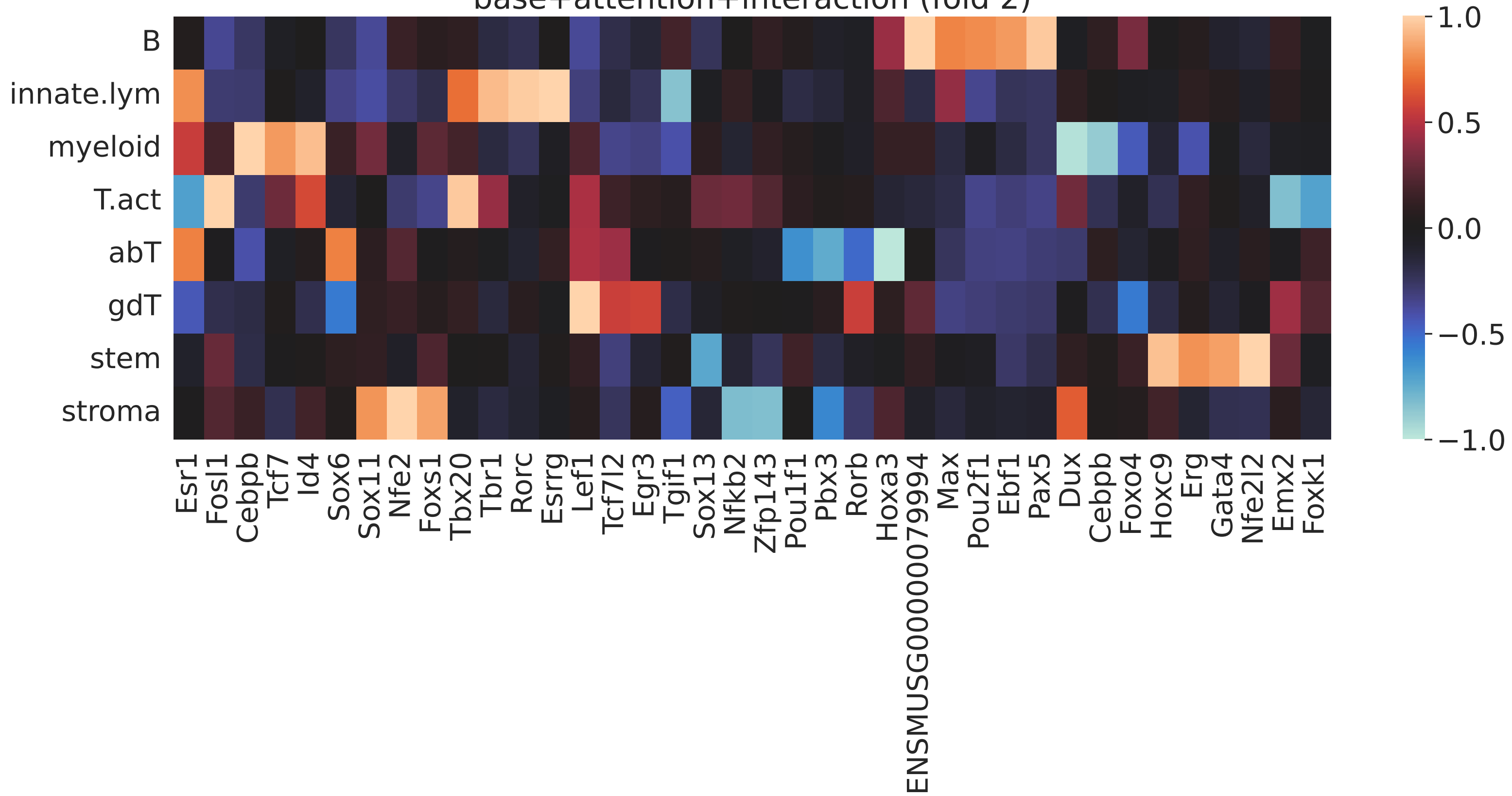

Supplement: btad271_Supplementary_Data [file btad271_supplementary_data.zip › supplementary/figures/Chikina.288.sup.4.pdf]

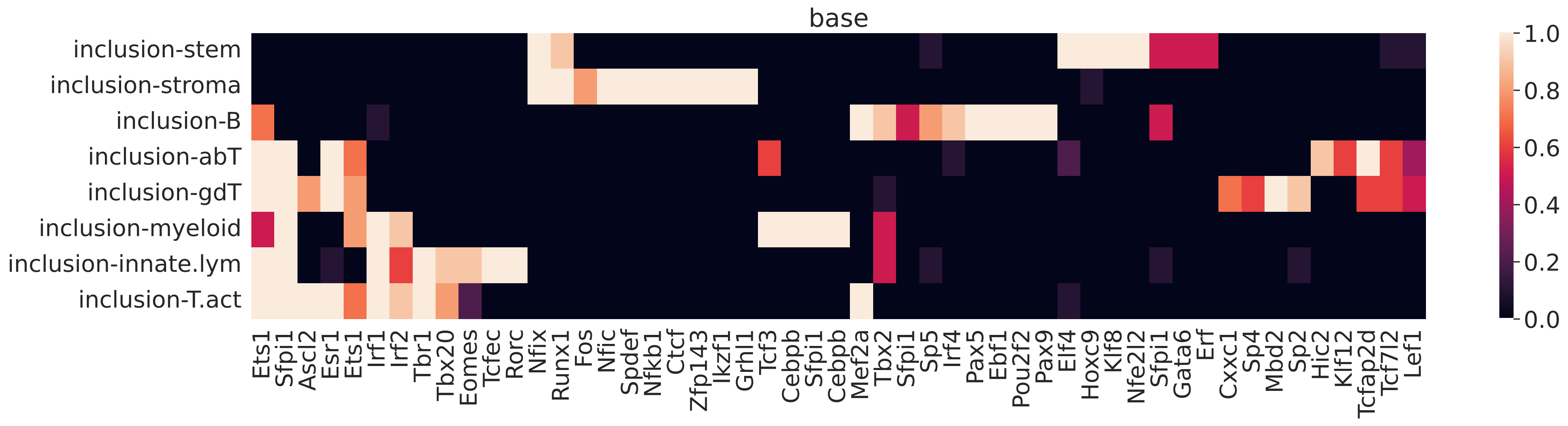

Supplement: btad271_Supplementary_Data [file btad271_supplementary_data.zip › supplementary/figures/Chikina.288.sup.5.pdf]

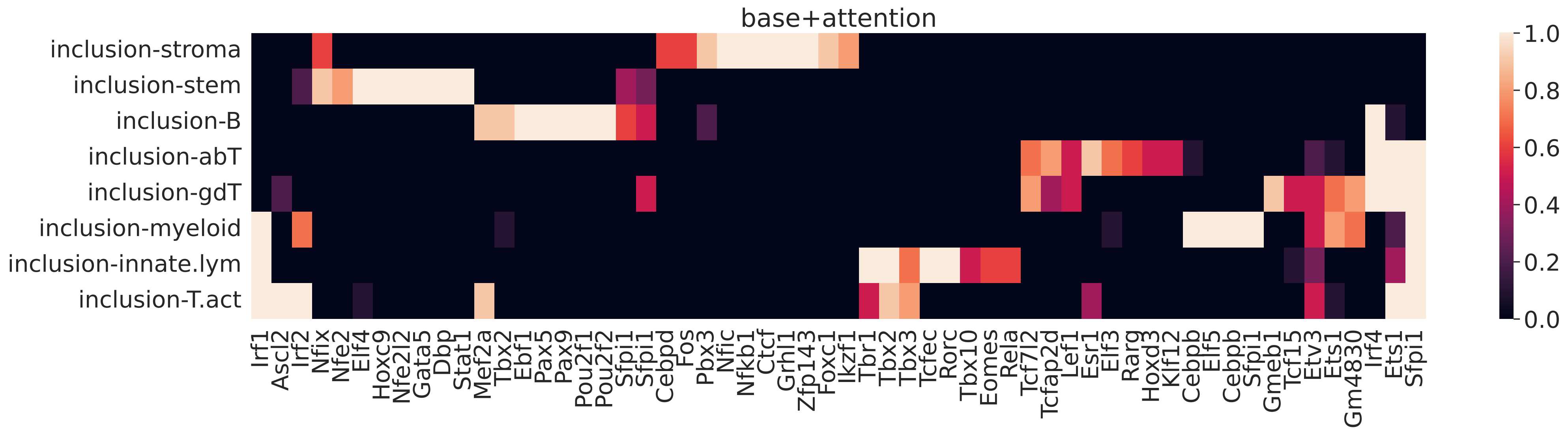

Supplement: btad271_Supplementary_Data [file btad271_supplementary_data.zip › supplementary/figures/Chikina.288.sup.6.pdf]

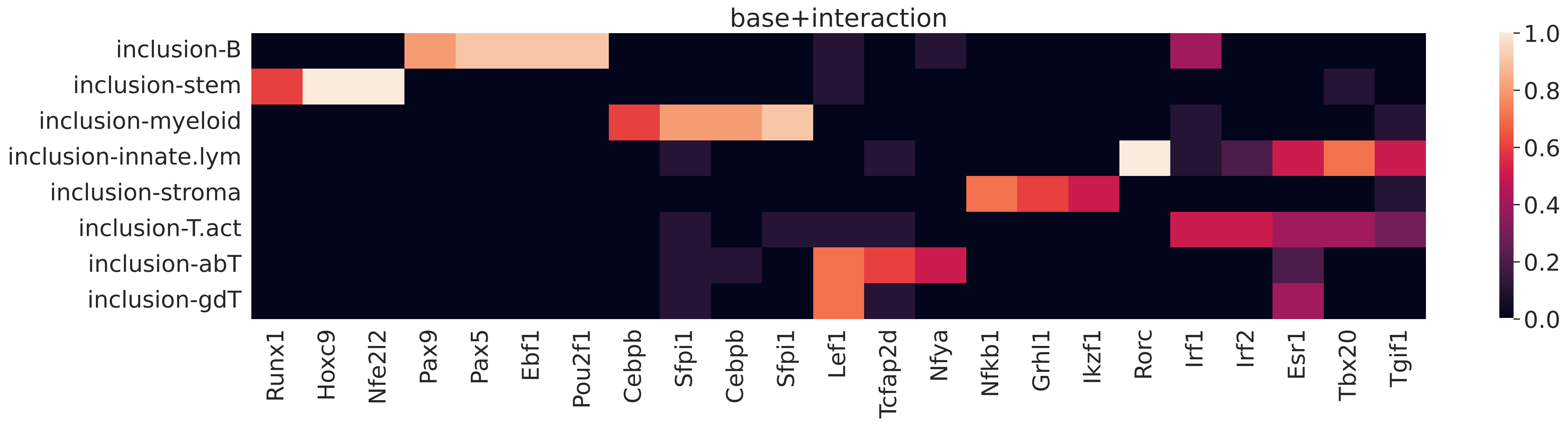

Supplement: btad271_Supplementary_Data [file btad271_supplementary_data.zip › supplementary/figures/Chikina.288.sup.7.pdf]

base+attention+interaction

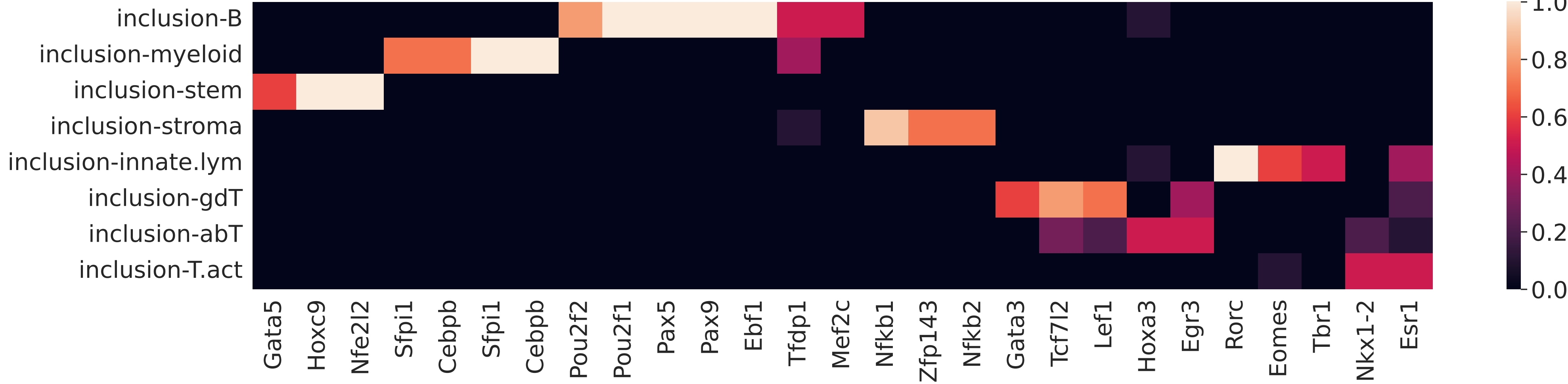

Supplement: btad271_Supplementary_Data [file btad271_supplementary_data.zip › supplementary/figures/Chikina.288.sup.8.pdf]

# cell-type abT

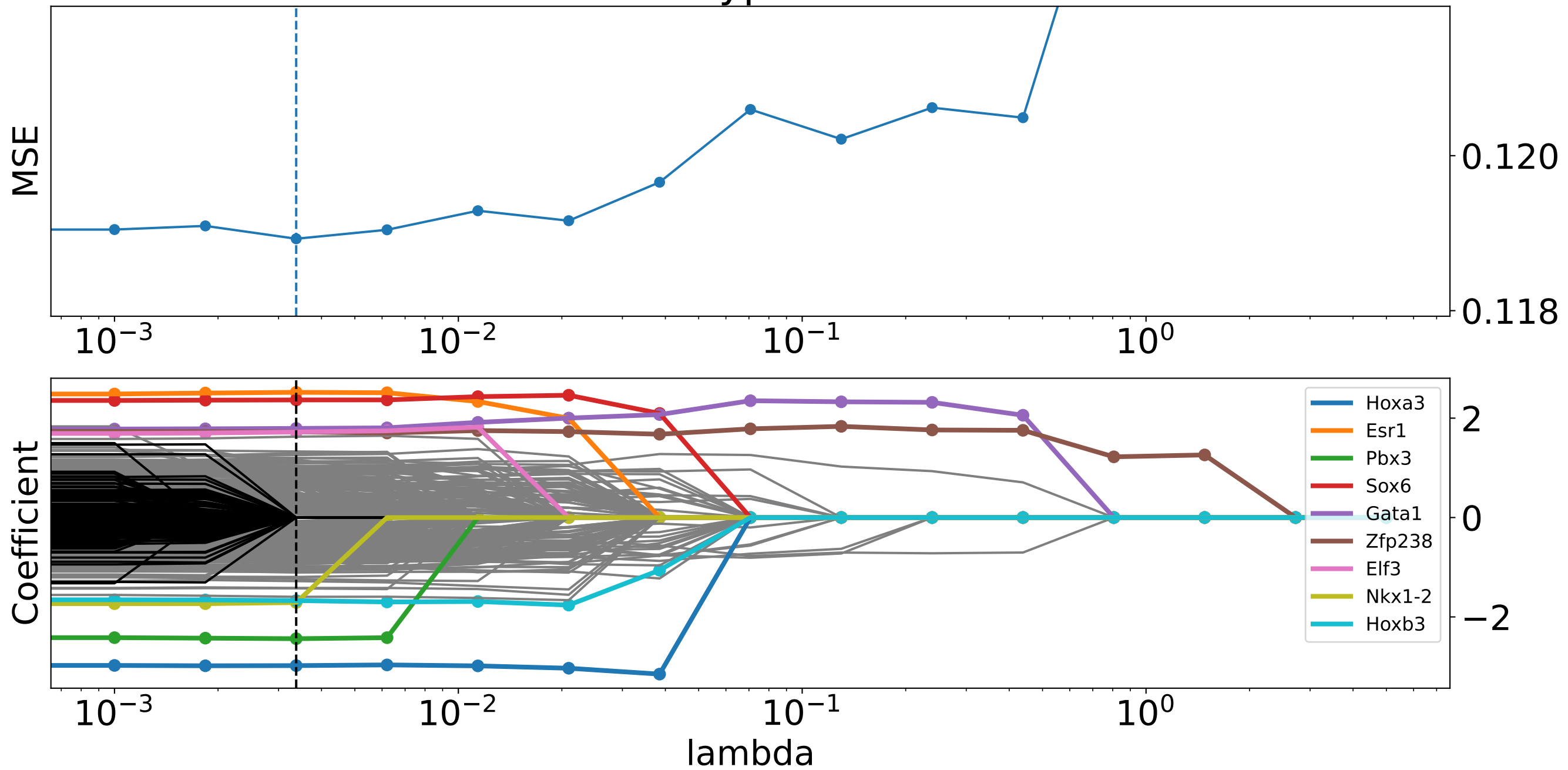

Supplement: btad271_Supplementary_Data [file btad271_supplementary_data.zip › supplementary/figures/Chikina.288.sup.9.pdf]

cell-type B

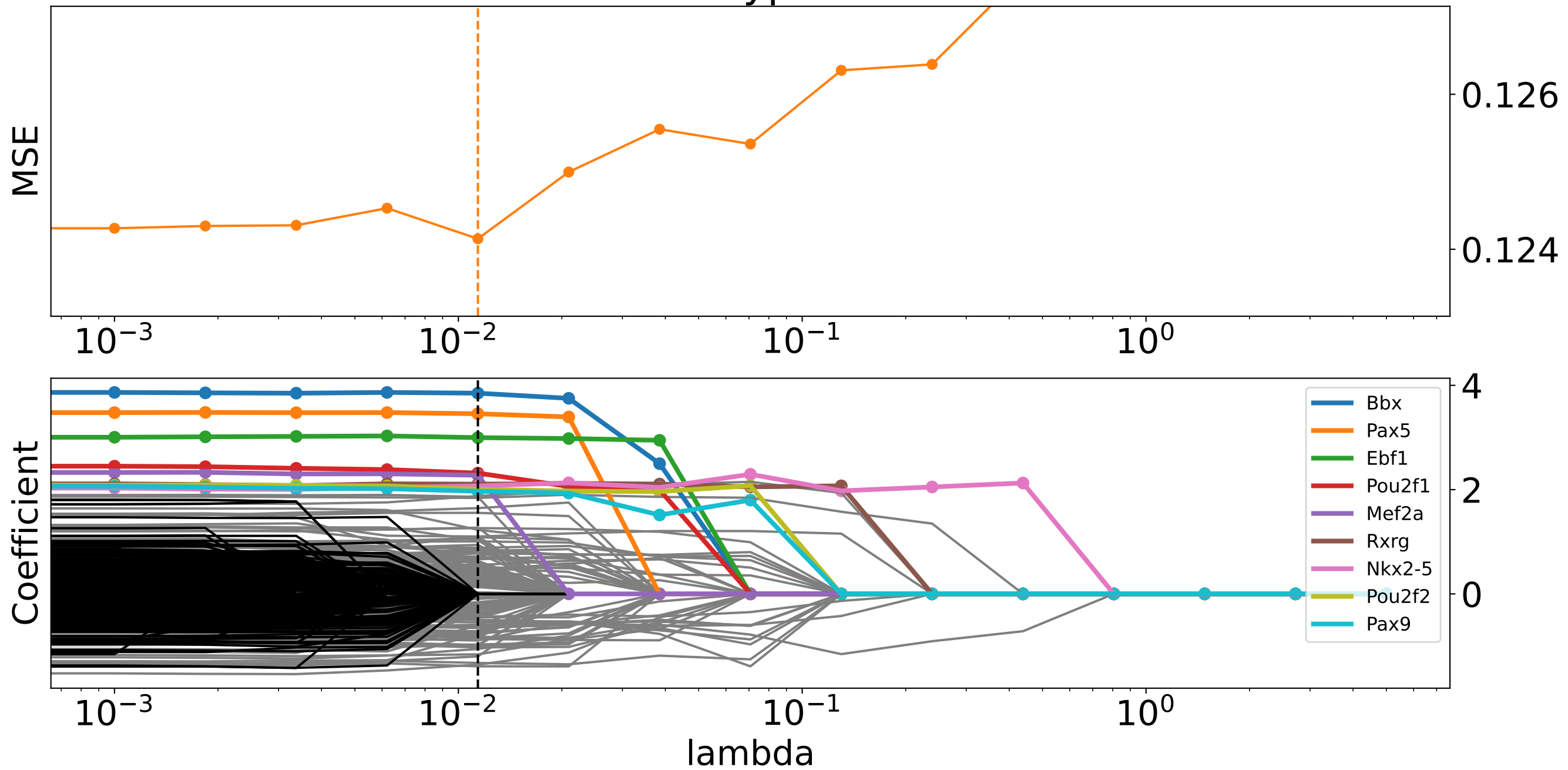

Supplement: btad271_Supplementary_Data [file btad271_supplementary_data.zip › supplementary/figures/Chikina.288.sup.10.pdf]

cell-type gdT

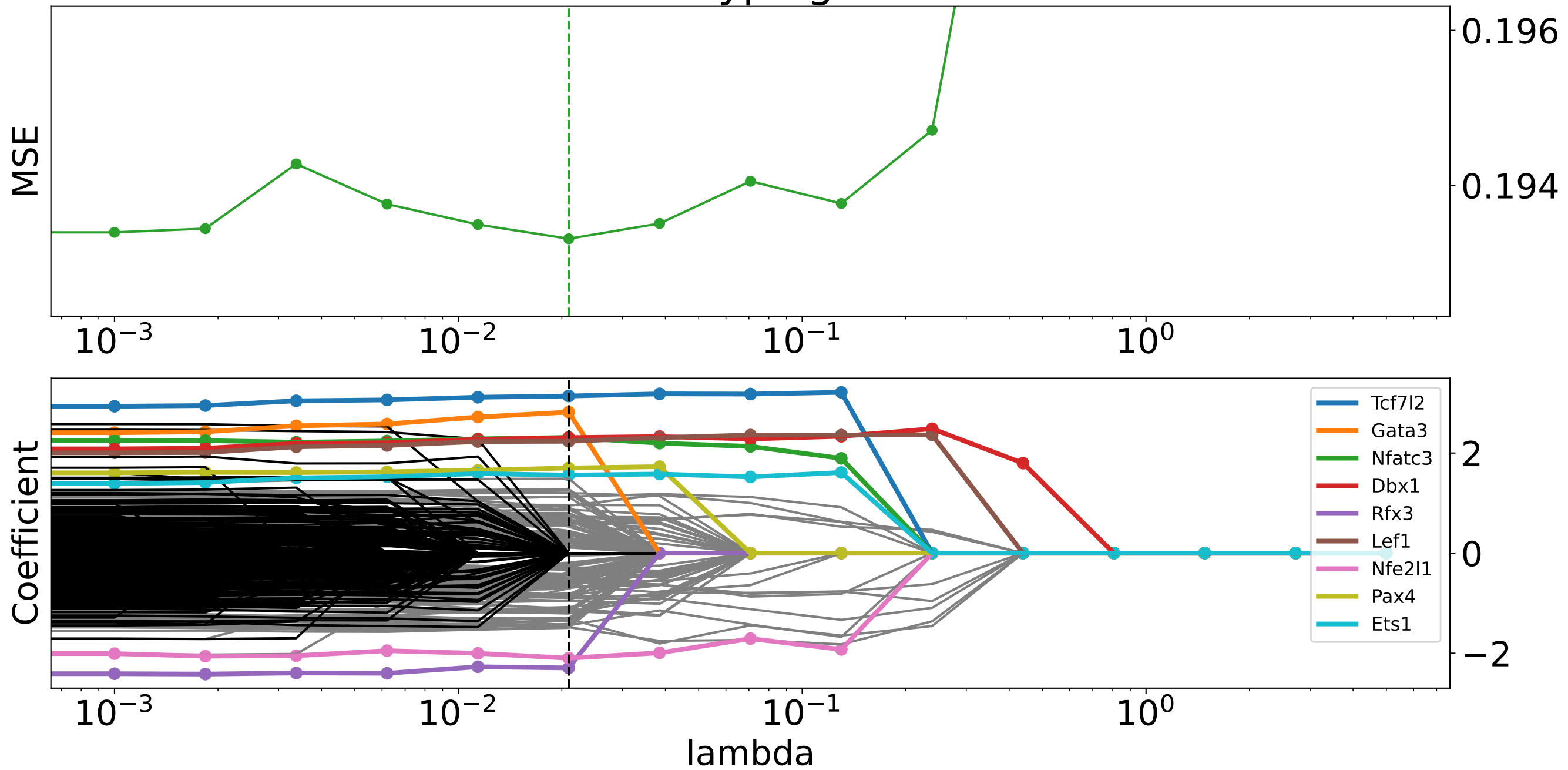

Supplement: btad271_Supplementary_Data [file btad271_supplementary_data.zip › supplementary/figures/Chikina.288.sup.11.pdf]

# cell-type innate.lym

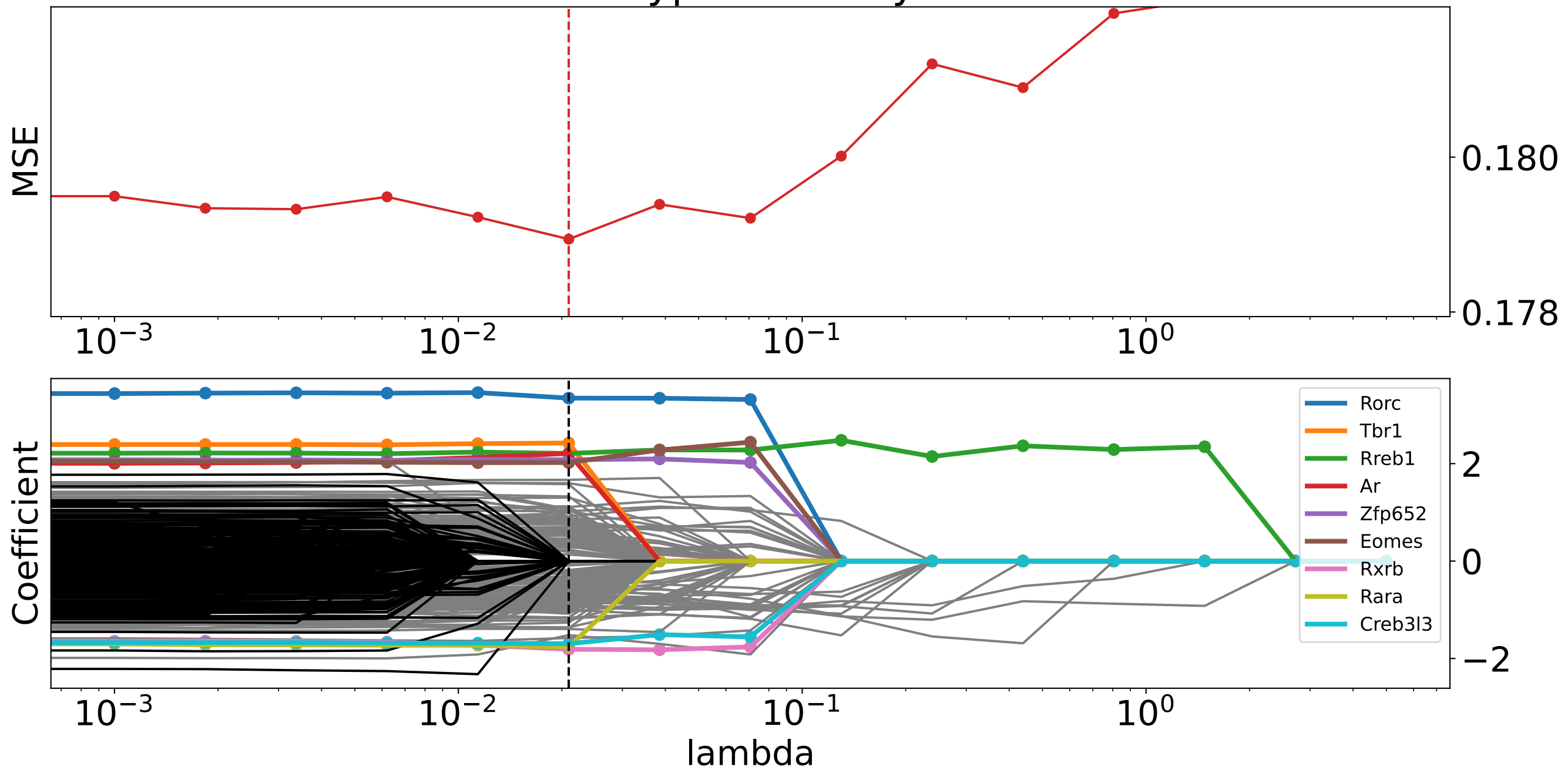

Supplement: btad271_Supplementary_Data [file btad271_supplementary_data.zip › supplementary/figures/Chikina.288.sup.12.pdf]

# cell-type myeloid

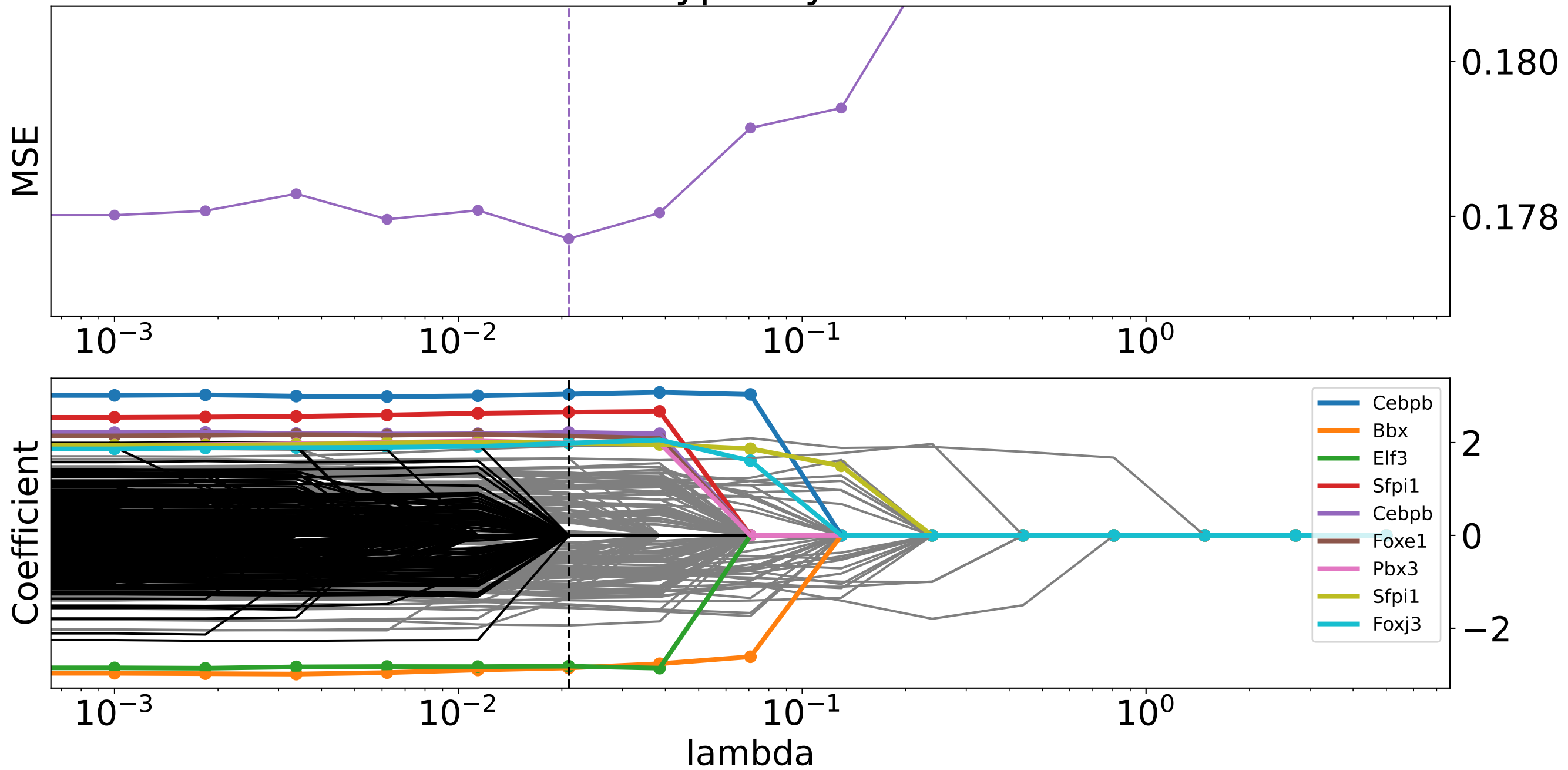

Supplement: btad271_Supplementary_Data [file btad271_supplementary_data.zip › supplementary/figures/Chikina.288.sup.13.pdf]

# cell-type stem

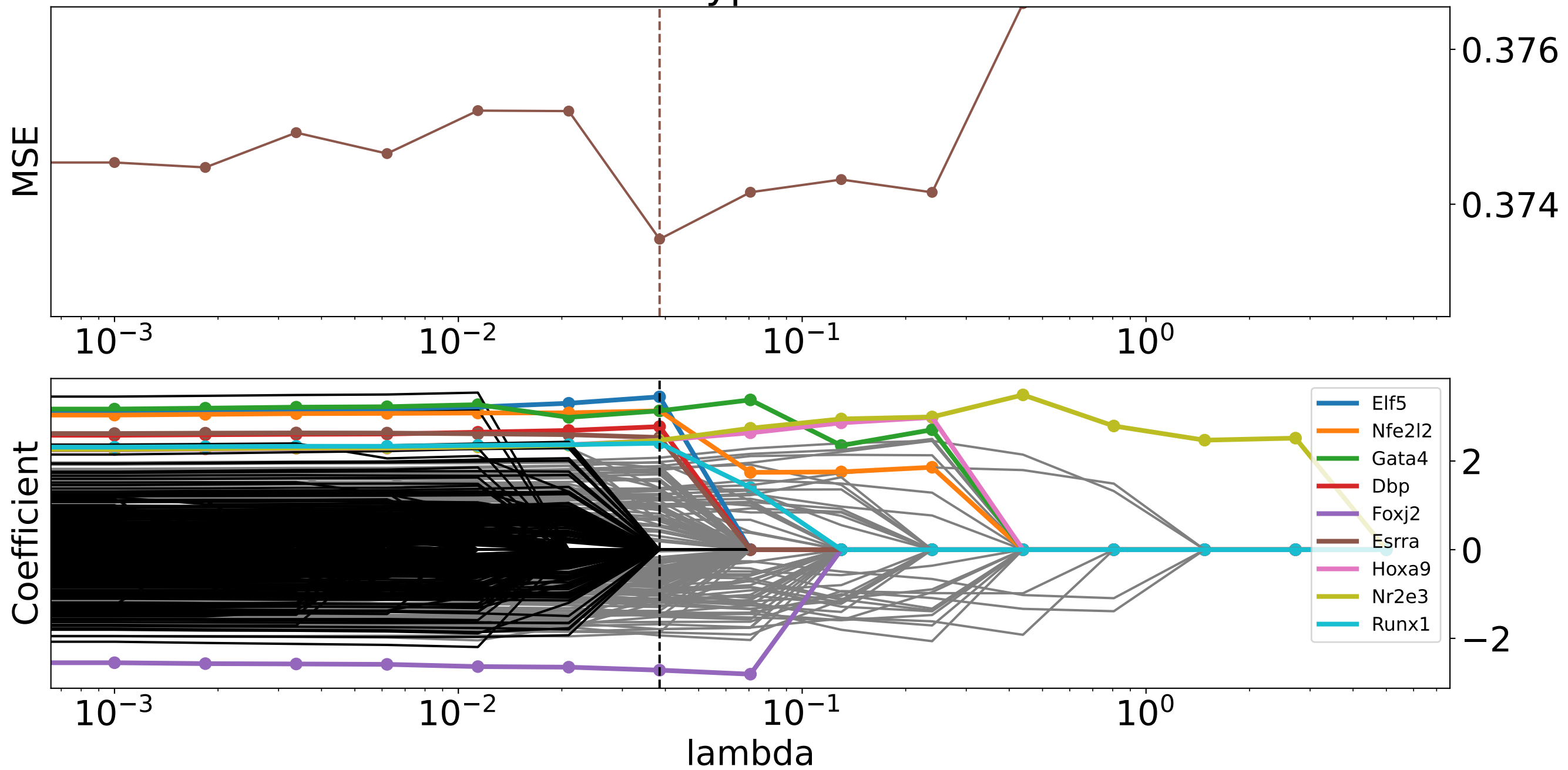

Supplement: btad271_Supplementary_Data [file btad271_supplementary_data.zip › supplementary/figures/Chikina.288.sup.14.pdf]

# cell-type stroma

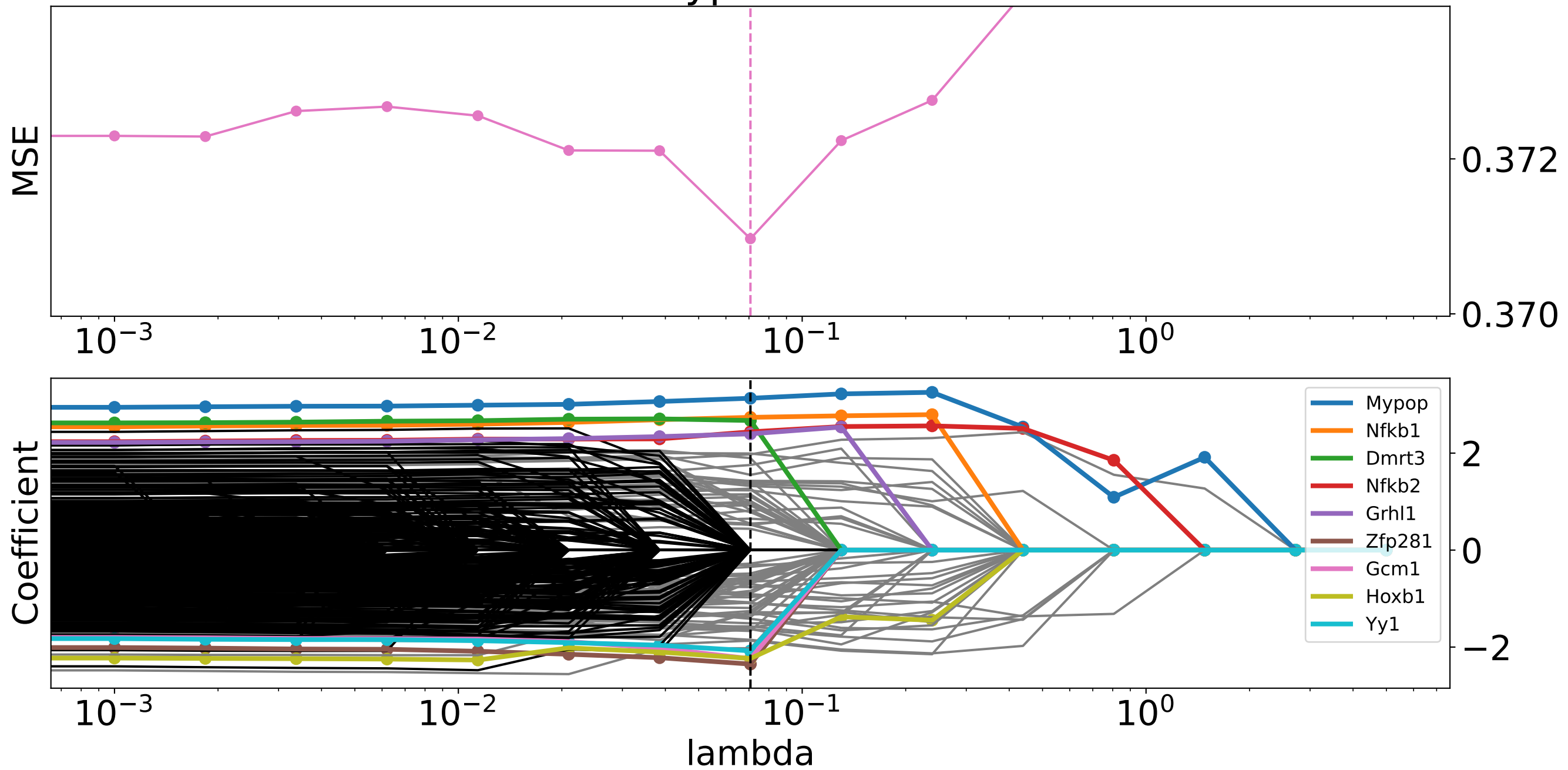

Supplement: btad271_Supplementary_Data [file btad271_supplementary_data.zip › supplementary/figures/Chikina.288.sup.15.pdf]

# cell-type T.act

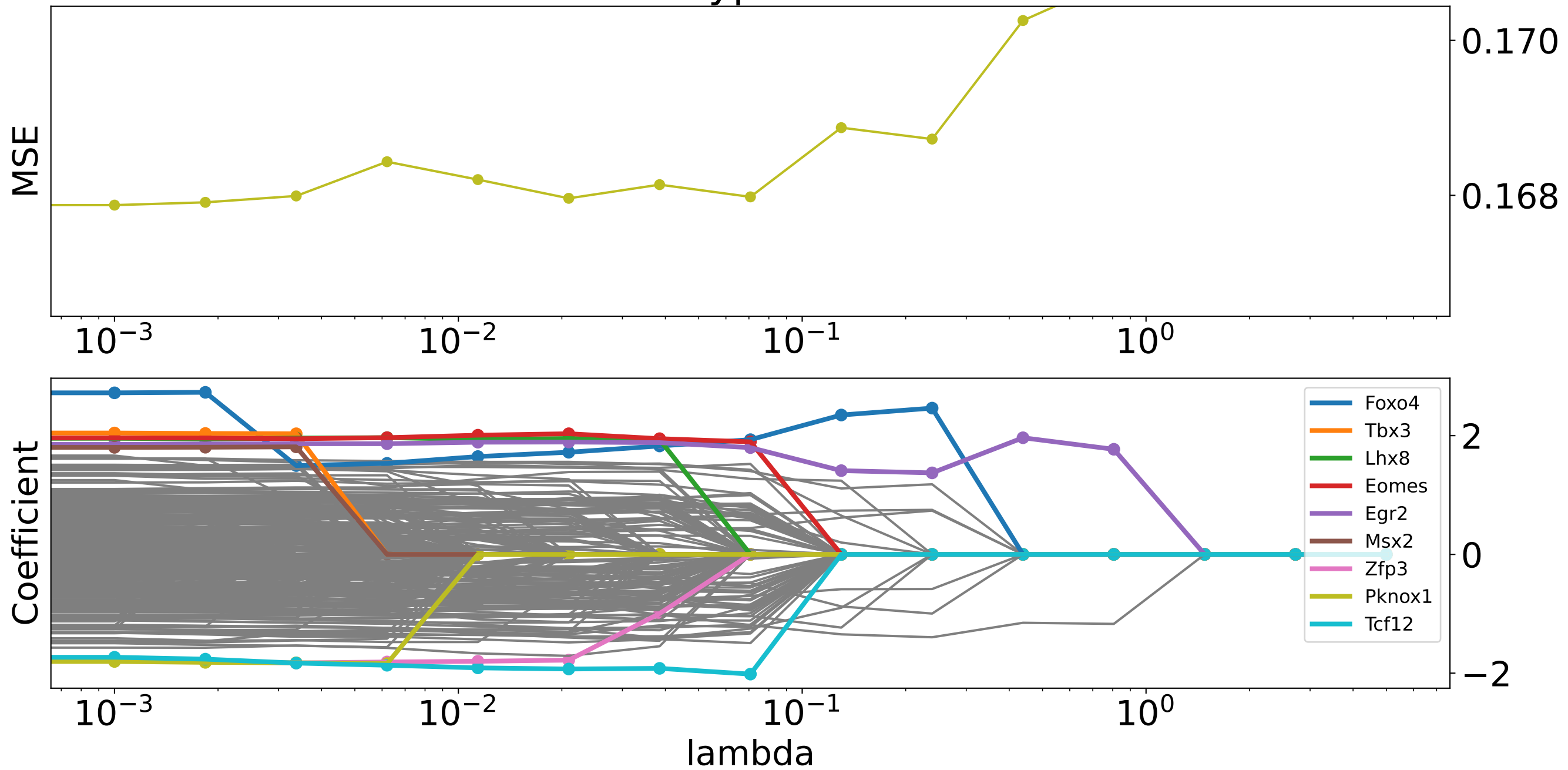

Supplement: btad271_Supplementary_Data [file btad271_supplementary_data.zip › supplementary/figures/Chikina.288.sup.16.pdf]

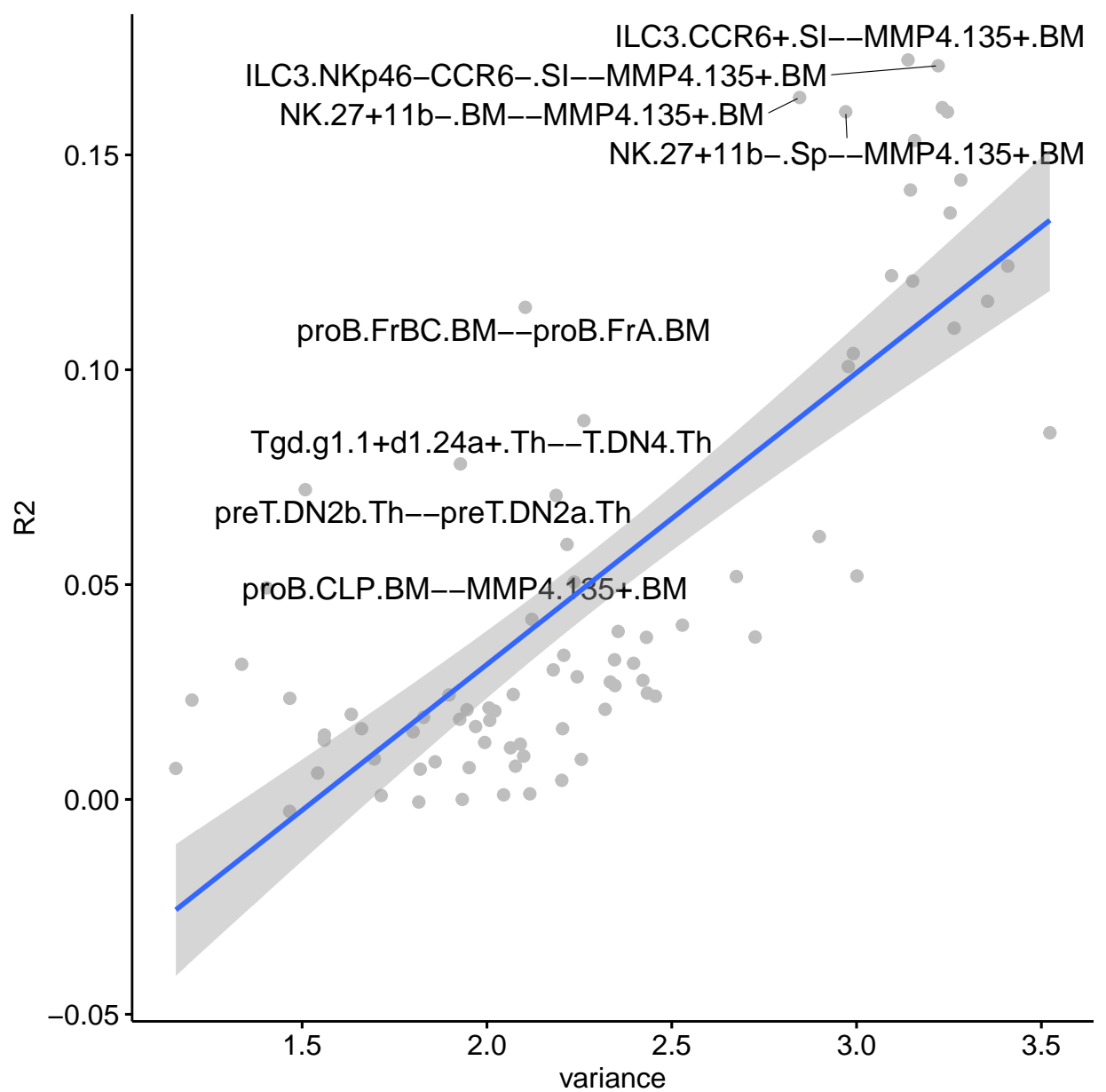

Supplement: btad271_Supplementary_Data [file btad271_supplementary_data.zip › supplementary/figures/Chikina.288.sup.17.pdf]
